# The Treatment of Varicocele

**Published:** 1893-11

**Authors:** 


### THE TREATMENT OF VARICOCELE.

In the *Gazette Hebdomadaire de Medicine et de Chirurgie*, March 25, 1893, Reclus discusses the treatment of varicocele. He is not an advocate of operation except in rare cases, such as when the swelling is excessively large, or provokes hypochondriasis, or threatens atrophy of the testicle. As conservative measure he orders a suspensory bandage, cold or very hot baths, rectal injections, and iodide of potassium or extract of hammamelis internally. Atrophy or even gangrene of the testicle may follow operative procedures. The cure is also not so complete as represented. the scrotum is often left lax and pendulous, and the tendency to venous stasis persist, and the trouble is apt to recur. For several years the author has resorted to excission of the scrotum, first practised by Dionis and afterwards introduced by Henry, of New York. The operation is done under cocaine anesthesia, a 1-per-cent. solution being employed. Particular stress is laid on the necessity of stopping all bleeding. He has done the operation in twenty-nine cases, with satisfactory results.—*Ex.*

